# Direct anterior total hip arthroplasty with dual mobility cup for femoral neck fractures in dementia patients

**DOI:** 10.1051/sicotj/2025034

**Published:** 2025-07-16

**Authors:** Ryuji Okuno, Tomonori Baba, Yu Ozaki, Yasuhiro Homma, Kazuo Kaneko, Muneaki Ishijima

**Affiliations:** 1 Department of Orthopedic Surgery, Juntendo University 2-1-1 Hongo Bunkyo-ku Tokyo Japan; 2 Department of Orthopedic Surgery, Juntendo Tokyo Koto Geriatric Medical Center 3-3-20 Shinsuna Koto-ku Tokyo Japan

**Keywords:** Total hip arthroplasty, Dual mobility cup, Femoral neck fracture, Dementia, Fracture mobility score

## Abstract

*Background*: Dementia patients with femoral neck fractures (FNFs) are unable to understand their dislocated limb positioning, which may impair rehabilitation and result in poorer functional recovery. Recently, good clinical results have been reported for the direct anterior approach for total hip arthroplasty (DAA-THA) using a dual mobility cup (DMC) for displaced FNFs. This study aimed to investigate differences in the clinical outcome of THA for displaced FNFs in patients with and without dementia. *Methods*: This study was retrospective and included 151 patients who underwent DAA-THA with DMC for displaced FNFs. Patients diagnosed with dementia prior to injury were classified into a dementia group (43 patients) and a non-dementia control group (control group, 108 patients). The evaluation items were age, sex, body mass index (BMI), preoperative Fracture Mobility Score (FMS), waiting period, preoperative anesthetic assessment, blood loss, operation time, complications, 1-year mortality, and 1-year FMS after surgery. The FMS was scored as: walking alone: 1, walking with a cane: 2, walking with a walker: 3, hand-guided walking: 4, and wheelchair: 5. *Results*: Significant differences were found in age, weight, BMI, and operation time. Postoperative dislocation was not observed in both groups. FMS was compared before and after injury in three categories: (1) unchanged from before injury, (2) one rank down, and (3) two or more ranks down. No significant differences were found in any of these categories (*p* = 0.09). Functional outcomes showed no significant difference in mobility recovery. The 1-year mortality rate was 9.35% (16 patients), with no significant difference between the two groups (*p* = 0.17). *Discussion*: DAA-THA using DMC for displaced FNFs may have similar functional outcomes and mortality rates in both patients with and without dementia.

## Introduction

As the global population ages, the incidence of hip fractures is increasing. The prevalence of hip fractures is predicted to double worldwide between 2035 and 2050 [1]. The World Health Organization reported in 1999 that the predicted US hip fracture population will be 650,000 by 2050 [2]. A previous study reported that at least one-third of patients with hip fractures have cognitive impairment [3]. Hip fractures are associated with decreased mobility and motor function, reduced quality of life, and high mortality [1]. The 1-year postoperative mortality rate for femoral neck fractures (FNFs) ranges from 8 to 23.4% [1, 4]. The complications of dementia can hinder postoperative recovery, including an inability to understand appropriate rehabilitation instructions and follow contraindications to prevent hip joint dislocation [3, 5]. In addition, dementia patients usually have less activity and poor self-care ability, which increases postoperative complications [6]. Therefore, a higher severity of dementia increases the mortality rate and worsens functional recovery [5].

Bipolar head arthroplasty (BHA) or total hip arthroplasty (THA) are widely accepted techniques for displaced FNFs [7]. Compared to BHA, THA is associated with better postoperative pain relief and functional outcomes, as well as a lower reoperation rate; however, the dislocation rate is higher [8]. The dual mobility cup (DMC), an implant specifically designed to prevent dislocation, and the direct anterior approach (DAA), which allows soft tissue preservation, are effective in THA for displaced FNFs [9]. We have reported good clinical results when DAA and DMC are combined in THA for displaced FNFs [7, 9]. DAA has the advantage of not only a reduced dislocation rate but also an early functional recovery due to soft tissue preservation, which may support faster recovery and improved postoperative autonomy [10].

We hypothesized that DAA-THA with a DMC would yield similar short-term outcomes in dementia and non-dementia patients. The purpose of this study was to investigate differences in the clinical outcome of THA for displaced FNFs with and without dementia complications.

## Materials and methods

### Subjects

**Figure 1:**
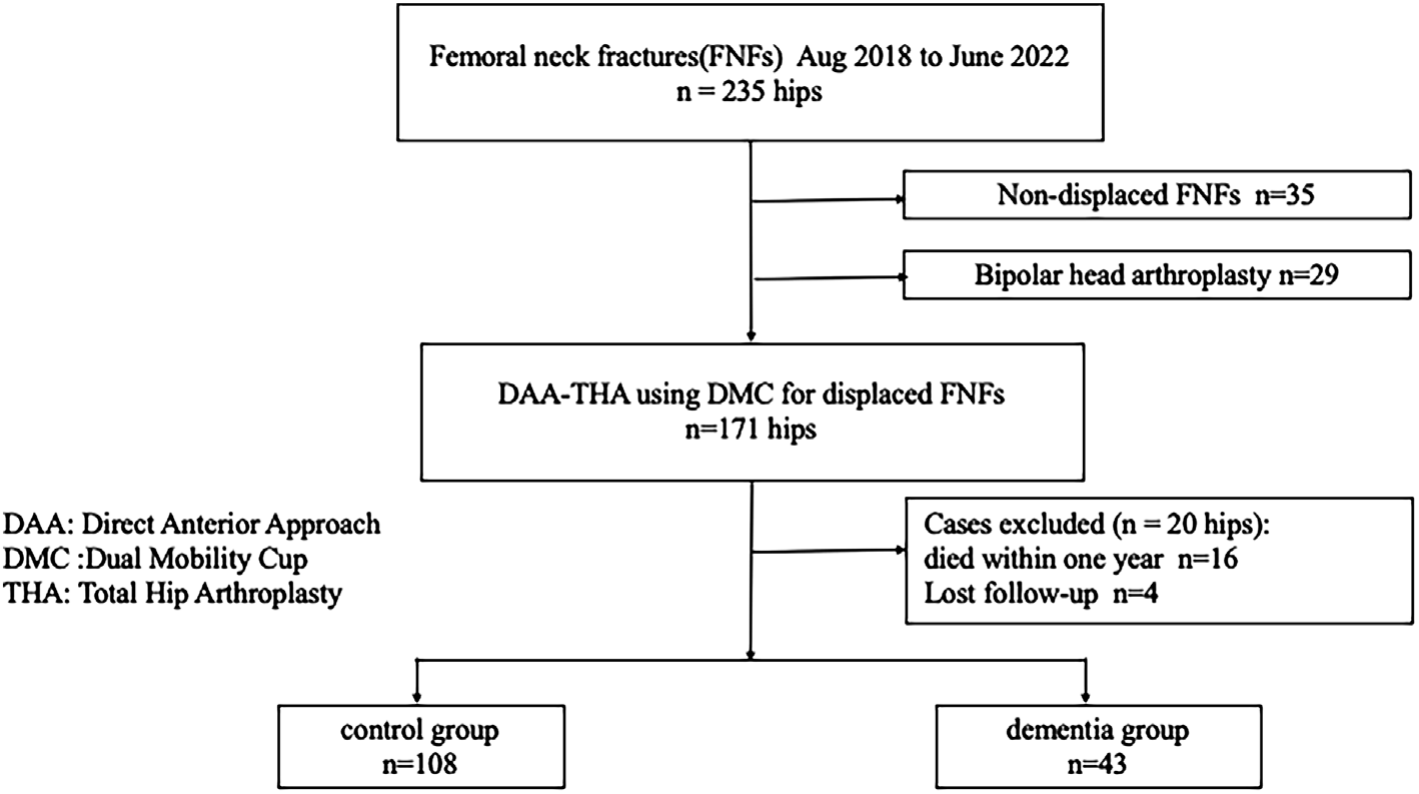
Flowchart.

The study was approved by the Hospital Ethics Committee of Juntendo University Hospital (E24-0260). Between August 2019 and June 2022, 235 FNFs were treated in our hospital at the two centers (Fig. 1, flowchart). Of these, 35 were non-displaced FNFs (all were treated with osteosynthesis) and 200 were displaced FNFs. Displaced FNFs were defined as follows: those with Garden classification types I and II with a posterior tilt of ≥20° and types III and IV [11]. We used computed tomography to confirm the extent of displacement. The indications for THA for FNFs at our institution were: (1) displaced type, and (2) patients were independent in activities of daily living (ADLs), including walking with a cane and walking with a gait aid, before the injury. We identified 29 patients whose ADLs were not independent (wheelchair or bedridden) before the injury and who had undergone BHA, and 171 patients who underwent DAA-THA with DMC. We excluded four patients lost to follow-up and 16 patients who died within one year (14 controls, 2 dementia). The study finally included 151 patients. Patients diagnosed with dementia before the injury were divided into a dementia group (43 patients) and a non-dementia control group (control group, 108 patients).

### Surgical procedure and implant information

Direct anterior approach was performed using a common fracture traction table or leg positioner (LECTURE; Surgical Alliance, Tokyo, Japan) with the patient in the supine position [12]. During implant insertion, the placement position was confirmed using intraoperative fluoroscopy. All patients started exercise therapy the day after surgery with no load restrictions.

Two different models of the Dual Mobility System were used: type 1 (Stryker, Mahwah, NJ) with Trident I or II and Modular Dual Mobility metal liners and type 2 (Zimmer-Biomet, Warsaw, IN) with G7 and E1 Active Articulation bearing. Details on stem types are summarized in Supplementary material.

### Evaluation

The evaluation items were age, sex, body mass index (BMI), preoperative Fracture Mobility Score (FMS), waiting period, preoperative anesthetic assessment, blood loss, operation time, complications, 1-year mortality, and 1-year FMS after surgery.

### Fracture Mobility Score (FMS)

In large clinical hip fracture audits, ongoing efforts are made to maintain the registration load as low as possible [13]; thus, simplification of queries is helpful and necessary to ensure that the value and reliability of the answers are not affected [14]. The Parker Mobility Score (PMS), comprising three questions, is a valid and reliable score to measure mobility in hip fractures [15]. Recently, the FMS, which has a strong correlation with the PMS [14], has been simplified to one question. It is scored as: walking alone: 1, walking with a cane: 2, walking with a walker: 3, hand-guided walking: 4, and wheelchair: 5.

### Analysis

The two groups were compared using an independent samples Student’s t-test for continuous variables, and a chi-squared test for dichotomous variables. The Mann-Whitney U test was used for nonparametric data and ordinal variables (including FMS) in independent groups. A value of *p* < 0.05 was considered significant.

## Results

Patient characteristics and operation information are summarized in Table 1. Significant differences were found in age, weight, BMI, and operation time. Cemented stems were used in 40 cases (26%) and cementless stems in 111 cases (74%). Regarding complications, periprosthetic femoral fractures were observed in three patients (two controls, one dementia), all with cementless stems; however, dislocation 1 year after surgery was not observed in either group (Table 2).

**Table 1:** Patient characteristics.

	Control (*n* = 108)	Dementia (*n* = 43)	*p*-Value
Sex (male/female)	25/83	12/31	0.725
Age	77.18 (8.78)	82.19 (7.69)	*<0.001
Height (cm)	155.1 (8.25)	153.6 (9.18)	0.35
Weight (kg)	50.4 (11.4)	44.8 (9.44)	*0.002
BMI	20.95 (4.32)	18.84 (3.34)	*0.001
Waiting period (day)	5.64 (3.49)	5.07 (2.49)	0.26
ASA classification			
Class 1	3	0	
Class 2	93	38	
Class 3	12	5	
Operation time (min)	114.2 (80.0)	93.4 (21.9)	*0.01
Blood loss (mL)	284.7 (135.5)	259.1 (100.6)	0.2
Cementless stem	79	32	
Cement stem	29	11	0.873

*Significant difference. Data: Average (SD). BMI, body mass index; ASA, American Society of Anesthesiologists. The bold values indicate statistically significant differences between the two groups (*p* < 0.05).

**Table 2:** Peri-operative complications for both groups.

	Control	Dementia
Periprosthetic femoral fracture	2	1
Great trochanter fracture	0	1
Subsidence (no fracture)	1	0
Dislocation	0	0
Infection	1	0
Aspiration pneumonia	1	3
Urinary infection	4	0
Diverticular hemorrhage	1	1
Intestinal ileus	1	0
Total (%)	10.2% (11/108)	14.0% (6/44)

FMS was compared before and after surgery in three categories: (1) unchanged from before injury, (2) one rank down, and (3) two or more ranks down (Table 3). No significant differences were found in any of these categories (*p* = 0.09). The 1-year mortality rate was 9.35% (16 patients), with no significant difference between the two groups (Table 4, *p* = 0.17).

**Table 3:** FMS was compared before and after surgery in three categories.

FMS	Control	Dementia
Unchanged	83 (76.9%)	29 (67.4%)
One rank down	16 (14.8%)	5 (11.6%)
Two or more ranks down	9 (8.3%)	9 (21.0%)

FMS: fracture mobility score. *p*-Value = 0.09.

**Table 4:** The 1-year mortality rate for both groups.

	Control	Dementia	*p*-Value
1-year mortality (%)	11.5 (14/122)	4.4 (2/45)	0.17

## Discussion

Patients with FNFs are at a higher risk for dislocation than patients with osteoarthritis, because of a combination of muscular insufficiency, cognitive and neurologic disorders, and recurrent falls that characterize patients with FNFs [16]. Previous studies stated that the dislocation rate in patients treated with hemiarthroplasty (HA) for FNFs was 3.8% compared with 10.7% in those treated with THA [17]. Therefore, prevention of dislocation is important in THA for FNFs, regardless of dementia. Due to cognitive limitations, dementia patients may struggle to avoid positions that risk dislocation postoperatively; thus, they are at a higher risk for dislocation. However, performing DAA with DMC had a strong preventive effect on dislocation, and no cases of dislocation were seen one year after surgery. The use of a minimally invasive muscle-tendon-preserving technique with DAA enabled early post-operative weaning; moreover, scoring of walking ability using FMS showed that dementia patients maintained the same walking ability as the controls after surgery. Conversely, when examined individually, the dementia group tended to show a greater deterioration in categories where FMS decreased by two or more ranks. The background is summarized in Table 5. The reason for this is thought to be that many dementia patients were transferred to facilities or mental hospitals after surgery instead of rehabilitation hospitals, where they did not receive active rehabilitation, resulting in a decline in their walking ability. These findings suggest that good outcomes can be achieved for FNFs in dementia patients by considering surgical technique and implant selection for hip arthroplasty.

**Table 5:** The trend in patients with two or more ranks down FMS.

	Control (*n* = 9)	Dementia (*n* = 9)	*p*-Value
Sex (male/female)	2/7	4/5	
Age	76.7	83.2	0.19
Height (cm)	154.6	155.6	0.83
Weight (kg)	43.8	44.56	0.87
BMI	18.4	18.24	0.93
Waiting period (day)	6.11	5.56	0.59
Operation time (min)	114.3	99.9	0.29
Blood loss (mL)	269.7	245.9	0.56
Admission to facility or mental hospital	2	7	
Neuromuscular disease	4	0	
No special remarks	3	2	

The treatment of dementia patients with FNFs is controversial. A previous study reported that dementia patients have low functional demands; therefore, less invasive methods of internal fixation should be considered [18]. Performing arthroplasty in dementia patients who are at a high risk of falls may also contribute to the development of post-operative complications such as peri-implant fractures and dislocations. However, as the population ages, the number of relatively independent dementia patients is expected to increase. We propose that minimally invasive osteosynthesis should not be chosen solely because the patient has dementia [18], and that the patient’s quality of life should be maintained.

Single mobile THA for FNFs in dementia patients increases the hospital length of stay, medical and prostheses-related complications, and cost of care compared with other therapeutic options [19]. Page et al. examined the dislocation rate of HA and reported that the dislocation rate was approximately three times higher in a cognitively impaired group compared with a cognitively intact group (3.4% vs. 1.3%); moreover, the posterior approach had a significantly higher dislocation rate than the anterolateral approach [20]. In addition, they reported that the risk of death was 3.2-fold higher in patients who experienced dislocation than in patients who did not, and dislocation effectively increased the risk of death by 221%. In recent studies, DMC-THA is reported to provide more benefits than HA in the treatment of FNFs [21, 22]. However, dementia patients are often excluded from surveys. To the best of our knowledge, there are two reports of THA using DMC for FNFs in dementia patients, consisting of the direct lateral approach [23] and posterolateral approach [24]. The present study is the first report of DAA, and there have been no previous reports comparing the clinical outcomes of dementia patients and healthy controls. DAA-THA using DMC appears to be a safe and effective option for displaced FNFs in dementia patients. Postoperative dislocation and reoperation in these previous studies were comparable to our results. The overall 1-year mortality rate in the present study was 9.3%, which is similar to the findings of previous studies [1, 4]. Further, there was no significant difference in the 1-year mortality rate between the dementia group (Table 6) and the direct lateral approach group (*p* = 0.18), but there was a significant difference from the posterolateral approach group (*p* = 0.0005). Anders et al reported that for the treatment of vulnerable elderly people with dementia who have a low activity level and short remaining time of life, factors such as the risk of polyethylene wear and associated aseptic loosening were of minor importance [24]. For such patients, pain-free joints and a decreased risk of dislocations, revision surgery, and readmission were more important; therefore, DMC-THA was considered favorable in the treatment of patients with displaced FNF and dementia patients [24]. Our findings support the use of DMC-THA for the treatment of such patients.

**Table 6:** Summary of the literature comparing THA using DMC for FNFs in dementia patients.

	Anders (2017)	Raffaele (2019)	Our study
Approach	Posterior lateral	Direct lateral	Direct anterior
Number	20	30	43
Age	83	82	82.19
ASA classification			
Class 2	8	3	38
Class 3	12	23	5
Class 4	0	4	0
Operation time	108	59	93.4
Dislocation	0	0	0
Reoperation	0	0	0
*One-year mortality (%)	45 (9/20)	13.3 (4/30)	4.4 (2/43)

*p < 0.001.

In the hip fracture population, the ambulatory level at 2 weeks after surgery is a significant predictor of survivorship at 1 year [1]. We hypothesized that DAA, which is less invasive to soft tissue, may have contributed to enabling early post-operative weaning and early muscle recovery, which led to shorter lying time; reduced perioperative complications, such as aspiration pneumonia and urinary tract infections; and reduced mortality in the short term. The occurrence of dislocations also limits rehabilitation and ADLs. The mortality rate of elderly people who experience dislocation after THA is sixfold higher than that of those who do not experience dislocation in the same period [25]; thus, dislocation itself may affect mortality [5]. Therefore, combining DAA with DMC to prevent dislocation may reduce mortality in this patient group. This study has several strengths. First, it is a large cohort study of dementia patients who had both DAA-THA and DMC. Second, there were no cases of postoperative dislocation. Finally, this is the first report comparing outcomes between patients with and without dementia using this approach.

There are several limitations to this study. First, information collection may have been incomplete due to the retrospective nature of the study. However, we consider the present study to be of sufficient value because there have been no studies specifically on DAA-THA using DMC with FNFs in dementia patients. Second, the follow-up period was short. Although following dementia patients over longer periods is challenging, further long-term studies are necessary. Finally, there was insufficient analysis of the severity of dementia using tools such as the Mini Mental State Examination (MMSE) and the Short Portable Mental Status Questionnaire (SPMSQ) [3]. This is considered to be that this study was a retrospective study conducted at multiple facilities, making it difficult to standardize the survey. In conclusion, DAA-THA using DMC appears to be a safe and effective option for displaced FNFs in dementia patients, offering good one-year outcomes in terms of walking ability, dislocation rate, and mortality.

## Data Availability

The data that support the findings of this study are not publicly available due to patient privacy and ethical considerations. However, they may be made available by the corresponding author upon reasonable request and with appropriate institutional approvals.

## Supplementary material

*Table S1*. Implant data. The supplemental material is available at https://www.sicot-j.org/10.1051/sicotj/2025034/olm.

## References

[1] Cichos KH, McGwin Jr G, Boyd B, Arthroplasty for Hip Fracture Consortium, Ghanem ES (2023) Direct anterior approach total hip arthroplasty is associated with reduced 1-year mortality and surgical complications after femoral neck fracture. J Arthroplasty 38, 2347–2354. 37271240 10.1016/j.arth.2023.05.045

[2] Delmas PD, Fraser M (1999) Strong bones in later life: luxury or necessity? Bull World Health Organ 77, 416–422. 10361759 PMC2557681

[3] Mukka S, Knutsson B, Krupic F, Sayed-Noor AS (2017) The influence of cognitive status on outcome and walking ability after hemiarthroplasty for femoral neck fracture: a prospective cohort study. Eur J Orthop Surg Traumatol 27(5), 653–658. 27796582 10.1007/s00590-016-1873-9 PMC5486608

[4] Fu G, Li M, Xue Y, Wang H, Zhang R, Ma Y, Zheng Q (2021) Rapid preoperative predicting tools for 1-year mortality and walking ability of Asian elderly femoral neck fracture patients who planned for hip arthroplasty. J Orthop Surg Res 16, 455. 34271974 10.1186/s13018-021-02605-0 PMC8283892

[5] Tarazona-Santabalbina FJ, Belenguer-Varea Á, Rovira Daudi E, et al. (2015) Severity of cognitive impairment as a prognostic factor for mortality and functional recovery of geriatric patients with hip fracture. Geriatr Gerontol Int 15, 289–295. 25164866 10.1111/ggi.12271

[6] Bai J, Zhang P, Liang X, Wu Z, Wang J, Liang Y (2018) Association between dementia and mortality in the elderly patients undergoing hip fracture surgery: a meta-analysis. J Orthop Surg Res 13(1), 298. 30470251 10.1186/s13018-018-0988-6 PMC6260652

[7] Jinnai Y, Homma Y, Baba T, Zhuang X, Kaneko K, Ishijima M (2021) Use of dual mobility acetabular component and anterior approach in patients with displaced femoral neck fracture. J Arthroplasty 36(7), 2530–2535. 33744082 10.1016/j.arth.2021.02.056

[8] Blomfeldt R, Tornkvist H, Eriksson K, Soderqvist A, Ponzer S, Tidermark J (2007) A randomised controlled trial comparing bipolar hemiarthroplasty with total hip replacement for displaced intracapsular fractures of the femoral neck in elderly patients. J Bone Jt Surg 89, 160–165. 10.1302/0301-620X.89B2.18576 17322427

[9] Homma Y, Baba T, Ozaki Y, Watari T, Kobayashi H, Ochi H, Matsumoto M, Kaneko K (2017) In total hip arthroplasty via the direct anterior approach, a dual-mobility cup prevents dislocation as effectively in hip fracture as in osteoarthritis. Int Orthop 41(3), 491–497. 27837328 10.1007/s00264-016-3332-y

[10] Baba T, Shitoto K, Kaneko K (2013) Bipolar hemiarthroplasty for femoral neck fracture using the direct anterior approach. World J Orthop 4, 85. 23610757 10.5312/wjo.v4.i2.85 PMC3631957

[11] Kalsbeek J, van Walsum A, Roerdink H, Schipper I (2022) More than 20° posterior tilt of the femoral head in undisplaced femoral neck fractures results in a four times higher risk of treatment failure. Eur J Trauma Emerg Surg 48(2), 1343–1350. 33903934 10.1007/s00068-021-01673-5 PMC9001535

[12] Nakamura J, Hagiwara S, Orita S, et al. (2017) Direct anterior approach for total hip arthroplasty with a novel mobile traction table – a prospective cohort study. BMC Musculoskelet Disord 18, 49. 28137262 10.1186/s12891-017-1427-2 PMC5282798

[13] Voeten SC, Arends AJ, Wouters M, et al. (2019) The Dutch hip fracture audit: evaluation of the quality of multidisciplinary hip fracture care in the Netherlands. Arch Osteoporos 14, 28. 30825004 10.1007/s11657-019-0576-3 PMC6397305

[14] Voeten SC, Nijmeijer WS, Vermeer M, Schipper IB, Hegeman JH (2021) Validation of the Fracture Mobility Score against the Parker Mobility Score in hip fracture patients. Injury 51, 395–399. 10.1016/j.injury.2019.10.035 31668574

[15] Parker MJ, Palmer CR (1993) A new mobility score for predicting mortality after hip fracture. J Bone Joint Surg Br 75(5), 797–798. 8376443 10.1302/0301-620X.75B5.8376443

[16] Guyen O (2019) Hemiarthroplasty or total hip arthroplasty in recent femoral neck fractures? Orthop Traumatol Surg Res 105((1S)), 95–101. 30449680 10.1016/j.otsr.2018.04.034

[17] Canton G, Moghnie A, Cleva M, Kostoris FM, Murena L (2019) Dual mobility total hip arthroplasty in the treatment of femoral neck fractures: a retrospective evaluation at mid-term follow-up. Acta Biomed 90, 98–103. 10.23750/abm.v90i1-S.8070 PMC6503413 30715006

[18] van Dortmont LM, Douw CM, van Breukelen AM, et al. (2000) Outcome after hemi-arthroplasty for displaced intracapsular femoral neck fracture related to mental state. Injury 31(5), 327–331. 10775686 10.1016/s0020-1383(99)00304-6

[19] Ahluwalia SS, Lugo JD, Gordon AM, Golub IJ, Razi AE, Feliccia J, Kang KK (2023) The association of dementia on perioperative complications following primary total hip arthroplasty for femoral neck fractures. Eur J Orthop Surg Traumatol 33, 971–976. 35230544 10.1007/s00590-022-03236-9

[20] Page BJ, Parsons MS, Lee JH, et al. (2023) Surgical approach and dislocation risk after hemiarthroplasty in geriatric patients with femoral neck fracture with and without cognitive impairments – Does cognitive impairment influence dislocation risk? J Orthop Trauma 37(9), 450–455. 37053111 10.1097/BOT.0000000000002614

[21] Boukebous B, Boutroux P, Zahi R, Azmy C, Guillon P (2018) Comparison of dual mobility total hip arthroplasty and bipolar arthroplasty for femoral neck fractures: A retrospective case-control study of 199 hips. Orthop Traumatol Surg Res 104(3), 369–375. 29454973 10.1016/j.otsr.2018.01.006

[22] Ma H-H, Chou T-A, Pai F-Y, et al. (2021) Outcomes of dual-mobility total hip arthroplasty versus bipolar hemiarthroplasty for patients with femoral neck fractures: a systematic review and meta-analysis. J Orthop Surg Res 16(1), 152. 33627151 10.1186/s13018-021-02316-6 PMC7903652

[23] Iorio R, Iannotti F, Mazza D, Speranza A, Massafra C, Guzzini M, D’Arrigo C, Ferretti A (2019) Is dual cup mobility better than hemiarthroplasty in patients with dementia and femoral neck fracture? A randomized controlled trial. SICOT J 5, 38. 31674902 10.1051/sicotj/2019035 PMC6824440

[24] Graversen AE, Jakobsen SS, Kristensen PK, Thillemann TM (2017)No dislocations after primary hip arthroplasty with the dual mobility cup in displaced femoral neck fracture in patients with dementia. A one-year follow-up in 20 patients. SICOT J 3, 9. 28176672 10.1051/sicotj/2016050 PMC5297327

[25] Lamo-Espinosa JM, Gómez-Álvarez J, Gatica J, et al. (2021) Cemented dual mobility cup for primary total hip arthroplasty in elder patients with high-risk instability. Geriatrics (Basel) 6(1), 23. 33800068 10.3390/geriatrics6010023 PMC8005968

