# Supplementary material for: Direct anterior total hip arthroplasty with dual mobility cup for femoral neck fractures in dementia patients
Source: SICOT J. 2025 Jul 16;11:39. doi: 10.1051/sicotj/2025034 (PMC12266661; doi:10.1051/sicotj/2025034)
Supplement: Supplementary file 1 — Table S1. Implant data. [file sicotj-11-39-s1.pdf]

Supplement : Implant data

	Type 1(Stryker)	Type 2(Zimmer-Biomet)
Stem variation	Accolade II : 92	CMK :31
	Exeter V40 : 8	Microplasty :12
		Avenir : 4
	[Other implants]<br>・SL-PLUS MIA HA (Smith & Nephew)：1<br>・TriFit TS (Corin)：1<br>・twinSys (MATHYS)：2	
